# Improving Compliance with Lithium Monitoring Guidelines through a Nurse-Led Clinic: A Full Clinical Audit Cycle

**DOI:** 10.1192/j.eurpsy.2026.10431

**Published:** 2026-07-31

**Authors:** M. Ali, N. Quigley, D. Dolan

**Affiliations:** 1 Psychiatry; 2 Clinical Nursing, HSE North Dublin Mental Health Services, Dublin, Ireland

## Abstract

**Introduction:**

Lithium is a widely prescribed mood stabilizer for bipolar disorder, requiring regular monitoring due to its narrow therapeutic index and potential for thyroid and renal dysfunction. NICE guidelines recommend 6-monthly thyroid function tests (TFTs) and Urea and Electrolytes (U&E’s), documented therapeutic lithium levels, clinical review of results, and structured follow-up. A local review of adult community mental health services in an urban setting identified inconsistent compliance with these standards.

**Objectives:**

To assess compliance with NICE guidelines on lithium monitoring, introduce a nurse-led intervention, and re-audit to evaluate its outcome.

**Methods:**

This full clinical audit cycle involved 14 patients on lithium under the care of a general adult community mental health service. A spreadsheet was developed to record all the data and was then analyzed.

*Cycle 1:* Assessed adherence to monitoring standards using clinical records, MAISE (Mental Health Information System), and Health link (for lab results and GP communications) from July to December 2024.

*Intervention:* In January 2025, a nurse-led lithium monitoring clinic was launched, offering structured appointments, reminders, GP collaboration, and systematic documentation. Psychoeducation was provided to the team on the results after completion of cycle one.

*Cycle 2:* Re-evaluated the same cohort using identical methods from January to July 2025.

**Results:**

Cycle 1 fell short across all guideline standards, with deviations ranging from 21% to 36%. After the nurse-led intervention, Cycle 2 showed marked improvement, with all parameters reaching over 85% compliance. The largest improvement was in the documentation and clinical sign-off of investigations (+29.2%), demonstrating the benefit of structured, clinician-led oversight.

**Image 1:**
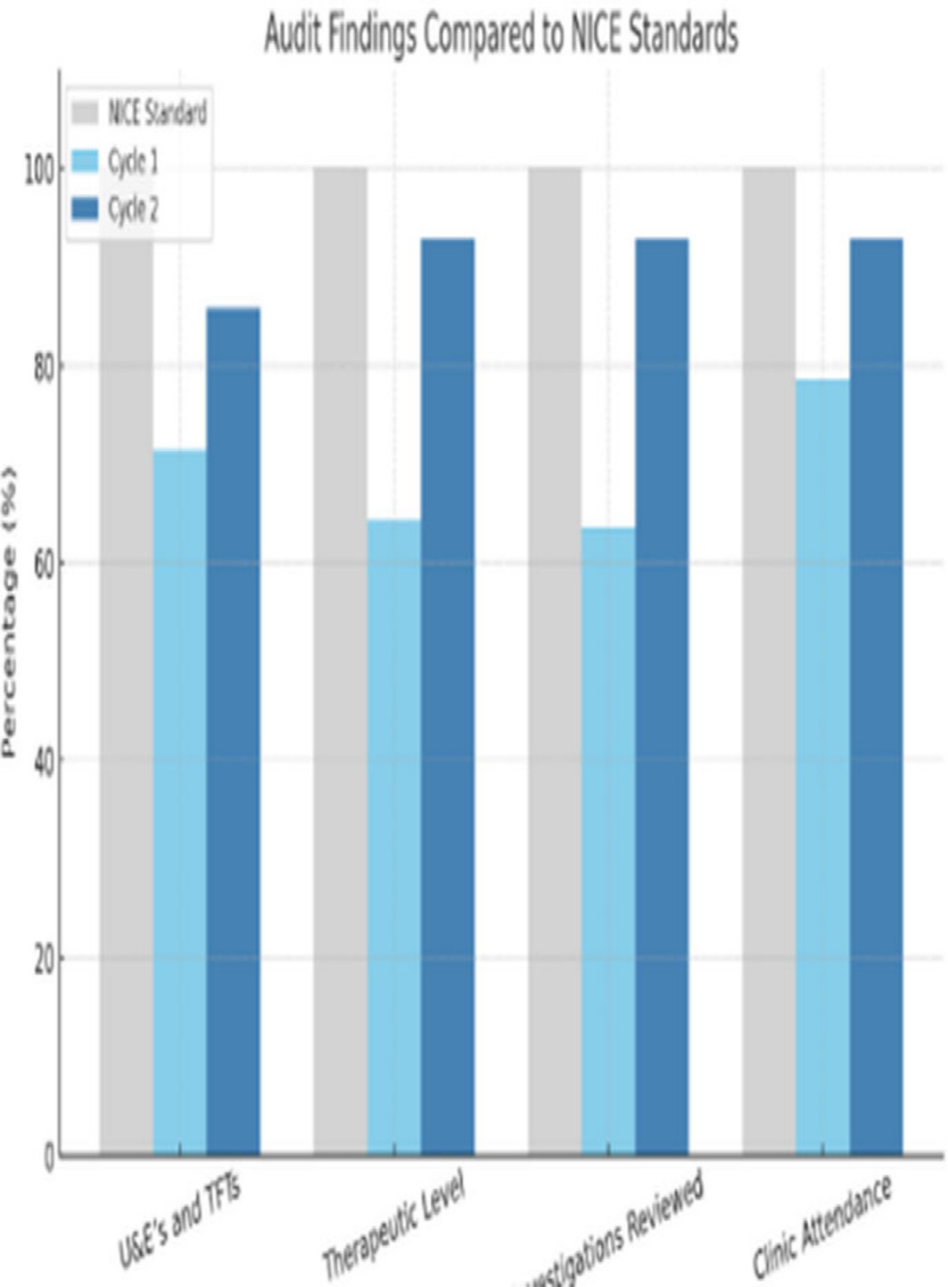
Image 1: Long description.

**Conclusions:**

The nurse-led lithium monitoring clinic significantly improved adherence to NICE standards, especially in ensuring biochemical monitoring and documented clinical reviews. This model demonstrates the effectiveness of structured service delivery in improving patient safety and care quality.

**Disclosure of Interest:**

None Declared

## Long descriptions

### Image 1: Long description

The y-axis represents Percentage (%) from 0 to 100. The x-axis lists four categories: U & E's and T F Ts, Therapeutic Level, Investigations Reviewed, and Clinic Attendance. Each category contains three bars: a light gray bar for NICE Standard (fixed at 100%), a light blue bar for Cycle 1, and a dark blue bar for Cycle 2.

* U & E's and T F Ts: Cycle 1 is at approximately 71% and Cycle 2 increases to approximately 86%.

* Therapeutic Level: Cycle 1 is at approximately 64% and Cycle 2 increases to approximately 93%.

* Investigations Reviewed: Cycle 1 is at approximately 63% and Cycle 2 increases to approximately 93%.

* Clinic Attendance: Cycle 1 is at approximately 79% and Cycle 2 increases to approximately 93%.

Across all categories, Cycle 2 shows a significant improvement over Cycle 1, moving closer to the 100% NICE Standard.

Navigate back to Image 1:.

